# The Importance Of Epigenetic Alterations In The Development Of Epstein-Barr Virus-Related Lymphomas

**DOI:** 10.4084/MJHID.2009.012

**Published:** 2009-11-15

**Authors:** Maria Takacs, Judit Segesdi, Ferenc Banati, Anita Koroknai, Hans Wolf, Hans Helmut Niller, Janos Minarovits

**Affiliations:** 1 Division of Virology, National Center for Epidemiology, H-1097 Budapest, Gyali út 2-6, Hungary; 2 Microbiological Research Group, National Center for Epidemiology, H-1529 Budapest, Pihenö u. 1. Hungary; 3 Institute for Medical Microbiology and Hygiene, University of Regensburg, Franz-Josef-Strauss-Allee 11, D-93053 Regensburg, Germany

## Abstract

Epstein-Barr virus (EBV), a human gammaherpesvirus, is associated with a series of malignant tumors. These include lymphomas (Burkitt’s lymphoma, Hodgkin’s disease, T/NK-cell lymphoma, post-transplant lymphoproliferative disease, AIDS-associated lymphoma, X-linked lymphoproliferative syndrome), carcinomas (nasopharyngeal carcinoma, gastric carcinoma, carcinomas of major salivary glands, thymic carcinoma, mammary carcinoma) and a sarcoma (leiomyosarcoma). The latent EBV genomes persist in the tumor cells as circular episomes, co-replicating with the cellular DNA once per cell cycle. The expression of latent EBV genes is cell type specific due to the strict epigenetic control of their promoters. DNA methylation, histone modifications and binding of key cellular regulatory proteins contribute to the regulation of alternative promoters for transcripts encoding the nuclear antigens EBNA1 to 6 and affect the activity of promoters for transcripts encoding transmembrane proteins (LMP1, LMP2A, LMP2B). In addition to genes transcribed by RNA polymerase II, there are also two RNA polymerase III transcribed genes in the EBV genome (EBER 1 and 2). The 5′ and internal regulatory sequences of EBER 1 and 2 transcription units are invariably unmethylated. The highly abundant EBER 1 and 2 RNAs are not translated to protein. Based on the cell type specific epigenetic marks associated with latent EBV genomes one can distinguish between viral epigenotypes that differ in transcriptional activity in spite of having an identical (or nearly identical) DNA sequence. Whereas latent EBV genomes are regularly targeted by epigenetic control mechanisms in different cell types, EBV encoded proteins may, in turn, affect the activity of a set of cellular promoters by interacting with the very same epigenetic regulatory machinery. There are EBNA1 binding sites in the human genome. Because high affinity binding of EBNA1 to its recognition sites is known to specify sites of DNA demethylation, we suggest that binding of EBNA1 to its cellular target sites may elicit local demethylation and contribute thereby to the activation of silent cellular promoters. EBNA2 interacts with histone acetyltransferases, and EBNALP (EBNA5) coactivates transcription by displacing histone deacetylase 4 from EBNA2-bound promoter sites. EBNA3C (EBNA6) seems to be associated both with histone acetylases and deacetylases, although in separate complexes. LMP1, a transmembrane protein involved in malignant transformation, can affect both alternative systems of epigenetic memory, DNA methylation and the Polycomb-trithorax group of protein complexes. In epithelial cells LMP1 can up-regulate DNA methyltransferases and, in Hodgkin lymphoma cells, induce the Polycomb group protein Bmi-1. In addition, LMP1 can also modulate cellular gene expression programs by affecting, *via* the NF-κB pathway, levels of cellular microRNAs miR-146a and miR-155. These interactions may result in epigenetic dysregulation and subsequent cellular dysfunctions that may manifest in or contribute to the development of pathological changes (e.g. initiation and progression of malignant neoplasms, autoimmune phenomena, immunodeficiency). Thus, Epstein-Barr virus, similarly to other viruses and certain bacteria, may induce pathological changes by epigenetic reprogramming of host cells. Elucidation of the epigenetic consequences of EBV-host interactions (within the framework of the emerging new field of patho-epigenetics) may have important implications for therapy and disease prevention, because epigenetic processes are reversible and continuous silencing of EBV genes contributing to patho-epigenetic changes may prevent disease development.

## Introduction:

Epstein-Barr virus (EBV), a member of the *Gammaherpesvirinae* subfamily, is a herpesvirus widespread in human populations^1^. EBV infects B lymphocytes *via* the complement receptor CD21, and the B-cell-bound virions can be transferred efficiently to CD21-negative epithelial cells^2^. Thus, when transmitted orally, EBV enters the epithelial cells of the oropharynx resulting in *productive (lytic) infection*. On the contrary, the EBV-B cell interaction is usually nonproductive, the *latent viral genomes* persist as circular episomes and co-replicate with the cellular DNA once per cell cycle.

The association of latent EBV genomes with malignant tumors is well documented. The virus has been discovered in cells derived from endemic Burkitt’s lymphoma samples^3^. In addition, other lymphomas (X-linked lymphoproliferative syndrome, posttransplant lymphoproliferative disease, AIDS-associated lymphoma, peripheral T/NK-cell lymphoma, Hodgkin’s lymphoma), certain carcinomas (nasopharyngeal carcinoma, gastric carcinoma, carcinomas of the major salivary glands, thymic carcinoma, mammary carcinoma) and a sarcoma (leiomyosarcoma) also regularly carry latent EBV genomes^1^,^4^ (Table 1).

Sequencing of the 172 kb double stranded linear genome of the prototype EBV strain (B95-8) significantly contributed to the identification of latent, growth-transformation associated proteins of the virus^5^.

**Figure 1.**
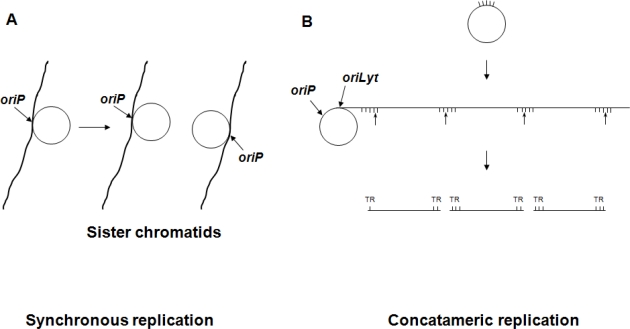
Alternative origins of EBV DNA replication: In **A**, the synchronous replication of latent EBV episomes and the cellular DNA (i.e. the chromosomal site where the episome is teethered by EBNA1) is demonstrated. EBNA1 (not shown on the figure) binds to *oriP*, the latent origin of EBV DNA replication, and it is anchored to a possibly AT rich chromosomal site as well. After replication, duplicated EBV episomes bind to the duplicated sites on adjacent sister chromatids (based on Nanbo et al.^10^). In **B**, concatemeric replication is initiated during the lytic cycle at *oriLyt.* After nuclease cleavage, double stranded linear EBV genomes with variable number of terminal repeats (TRs; their borders are indicated by vertical bars) are generated (based on Sato et al.^7^).

During productive infection all EBV encoded proteins are expressed and concatemeric replication of circular viral DNA templates is initiated at *oriLyt* (the lytic origin of replication) by the viral DNA polymerase with the help of EBV encoded transactivator and replication proteins^6^,^7^,^8^ (Figure 1)

**Figure 2.**
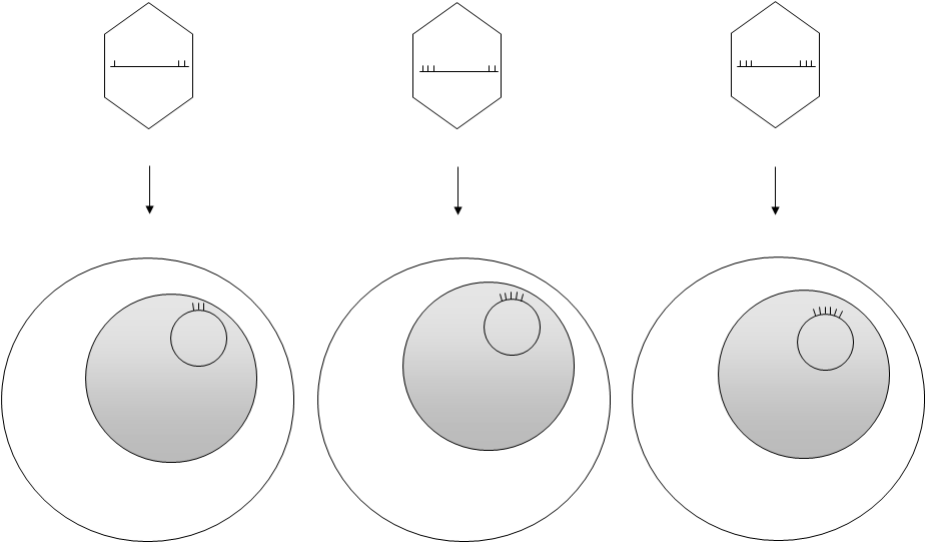
**Clonality of EBV-associated neoplasms:** The double stranded linear EBV genomes packaged into virions have variable numbers of terminal repeats (TRs, bordered by vertical bars on the figure) at their ends. After infection of cells, the termini fuse forming a circular episome harbouring distinct numbers of TRs. Clones of transformed cells can be characterized by determining the size of the *Bam*HI fragment carrying the TRs (see Raab-Traub and Flynn^11^).

The viral DNA packaged into the virion ends in terminal repetitions (TRs). Soon after *in vitro* infection of B cells the TRs fuse with each other and circular episomes are generated by amplification of a single copy of the viral DNA. Only a subset of the viral genes are transcribed from the latent EBV episomes that co-replicate with the cellular genome in the emerging B cell clones. It is noteworthy that during latent, non-productive infection, DNA synthesis is initiated at a distinct origin of replication called *oriP* (that is different from *oriLyt*), by the cellular DNA replication machinery^9^,^10^ (Figure 1). Expression of growth-transformation-associated viral latency genes leads to the establishment of permanently growing (immortal) lymphoblastoid cell lines (LCLs). The major EBV-associated malignancies also carry predominantly circular EBV DNA molecules. EBV infection is apparently important in their development, because analysis of fused TR sequences showed that these tumors are clonal proliferations of cells infected by EBV on a single occasion, presumably in an early stage of tumorigenesis^11^ (Figure 2).

## Host cell phenotype-dependent expression of latent EBV genes: EBV latency types:

The majority of EBV encoded proteins is involved in *productive EBV infection* and includes transactivators, enzymes necessary for viral DNA amplification and structural components of the virions. There is a sequential order of gene activation in the course of the lytic cycle: immediate early genes (BZLF1 and BRLF1) are expressed first and activate the so called early genes. Late proteins are expressed after viral DNA synthesis^12^.

**Figure 3.**
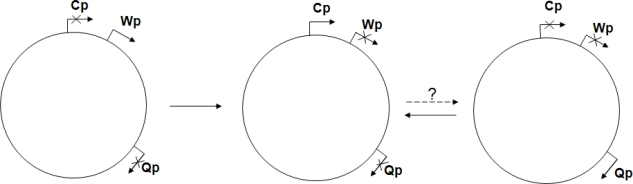
Promoter switch in latent EBV episomes: After initial EBV infection of B cells *in vitro*, Wp (a promoter located to the BamHI W fragment of the EBV genome) is switched on. A giant transcript coding for 6 nuclear antigens (EBNA1-6) is generated but the activity of Wp is transient. The transactivator protein EBNA2 and the EBNA1-bound *oriP* enhancer may contribute to switching on Cp, a B lymphoblast specific promoter. Burkitt’s lymphoma (BL) cells use a different promoter, Qp, to generate a transcript for EBNA1 only. *In vitro* cultivated BL cells may switch from Qp to Cp (arrow to the left). This promoter switch results in a phenotypic drift (from memory B cell to activated B cell phenotype). A Cp to Qp switch has been predicted but never observed (broken arrow) (see Niller et al.^13^).

A more restricted pattern of viral gene expression can be observed in *latently infected* cells. The latent EBV genomes carried by *in vitro* transformed LCLs and lymphoproliferations developing in recipients of organ or bone marrow transplants express six nuclear proteins (EBNAs). The EBNA1-6 transcripts are initiated at the C promoter (Cp, located to the *Bam*HI C fragment of the viral genome), hence the term *„Cp on” latency* (also called latency type III)^13^ (Table 2, Figure 3).

Cp is active only in B lymphoblasts^14^. EBNA1 is required for stable maintenance of viral episomes, whereas EBNA2, the major transactivator protein of EBV transactivates both viral promoters (Cp, LMP1p, LMP2Ap, LMP2Bp, see below) and promoters of cellular genes (CD23, CD21). EBNA3-6 modulate the transactivator activity of EBNA2 (see below).

In *„Cp on” latency* (reviewed by Niller et al.^1^) one can also detect three transcripts for latent membrane proteins (LMP1, LMP2A, LMP2B), two small RNA molecules (EBER 1 and 2), transcripts derived from the *Bam*HI A fragment of the genome (BARTs, reviewed by Smith^15^) and a series of microRNAs^16^,^17^,^18^. The transmembrane proteins LMP1 and LMP2 affect various signal transduction pathways. Expression of LMP1 in rodent cells causes malignant transformation. The EBERs and BARTs (and the protein products of BARTs), and the BARF1 protein were also implicated in oncogenesis (reviewed by Niller et al.^1^). The microRNAs derived from transcripts of the *BHRF1* gene and the BART transcripts may target both viral and cellular RNAs affecting thereby the abundance of lytic (BALF5) and latent (LMP1) EBV transcripts as well as certain cellular RNAs (e.g. that of CXCL-11, coding for a chemokine) (for review see Swaminathan^19^).

In other EBV-carrying cell types Cp is switched off (*„Cp off” latency*). These include memory B cells, Burkitt’s lymphomas (BLs) and BL cell lines that maintain the BL biopsy phenotype (type I BL cell lines), EBV-associated carcinomas (nasopharyngeal carcinoma, gastric carcinoma) and Hodgkin’s lymphomas. In *„Cp off latency”* typically an alternative promoter (Q promoter or Qp) is used for expression of EBNA1 transcripts, but the transcripts coding for the other five EBNAs are absent. The various cell types carrying silent C promoters may differ from each other regarding the expression of LMPs, BARTs, BARF1 and EBV-encoded microRNAs (latency types 0, I and II, Table 2, Figure 3)^1^, ^15^,^17^.

EBNA1 binds to *oriP*, the latent origin of EBV replication that acts as a long-range enhancer of Cp and LMP1p. This enhancer activity is essential for EBNA2 expression during immortalization of B cells *in vitro*^20^. In the absence of EBNA2 (the major viral transactivator protein in latency type III), the LMP promoters are activated by cellular proteins in nasopharyngeal carcinomas and in Sternberg-Reed cells of Hodgkin’s disease (latency type II), but not in type I BL cell lines or BL biopsies (type I latency) or memory B cells (type 0 latency, characterized by variable Qp activity)^1^.

It was observed by several laboratories that EBV latency promoter-reporter constructs were active after *in vitro* transfection into EBV carrying cell lines even if the corresponding latency promoter located on the viral episomes was silent^21^,^22^,^23^,^24^. This observations suggested that the transfected cells contained the transcription factors necessary for promoter activity and raised the possibility that silencing of latent viral promoters may be associated with their inaccessibility to cellular transcription factors. In the next part we briefly outline the epigenetic modifications and mechanisms potentially regulating promoter accessibility.

## Epigenetic modifications and epigenetic regulatory systems:

Modified bases in the DNA of prokaryotes may serve as identification marks that permit discrimination between self and foreign (invading) DNA. Well characterized endodeoxyribonuclease (restriction endonuclease) and DNA methyltransferase (modification methylase) activities, recognizing the same DNA sequence, enable bacterial cells to resist infections by phage or plasmid DNA molecules that are unmethylated or methylated at recognition sites different from those marked on the host DNA (reviewed in Wilson, 1998, Bujnicki, 2001, Murray, 2002). In addition to inhibiting the activity of restriction endonucleases, sequence-specific methylation may modulate (repress or activate) the expression of certain bacterial gene sets as well, influencing thereby bacterial virulence^25^,^26^. Modified bases are also involved in postreplicative repair processes in bacteria and may contribute to membrane binding and segregation of the chromosome as well^27^,^28^.

The role of base modification mediated by DNA methyltransferases apparently changed in large-genome eukaryotes. Bestor proposed that in these organisms DNA methyltransferases perform a new function, *the compartmentalization of the genome*^29^. This would provide an easy access to the unmethylated fraction of the genome by diffusible regulatory factors, while the usually larger, methylated fraction would remain inaccessible for them. One could argue that this novel function of DNA methyltransferases is reflected in the discontinuous, and changing methylation patterns of vertebrate genomes. In contrast to prokaryotes where the methyl-transferases modify essentially all of their recognition sites, in vertebrates there are alternating domains of methylated and unmethylated regions, and the methylation patterns of a certain region may change during development and differentiation (tissue-specific methylation patterns). The unmethylated domains, called CpG islands, are regularly found 5′ to coding sequences of constitutively expressed „housekeeping” genes^30^,^31^. It is important to note, however, that most CpG island promoters are unmethylated independently of their activity^32^,^33^. Thus, mechanisms other than DNA methylation may also silence CpG island promoters.

In mammals 5-methylcytosine (confined to the sequence 5′-CpG-3′, shortly: CpG) is the predominant modified base found in DNA^34^,^35^. Comparison of the amino acid sequence of DNA methyltransferase I (DNMT1), one of the mammalian DNA (cytosine-5)-methyltransferases, to the sequences of bacterial methyltransferases suggested that DNMT1 arose by fusion of a prokaryotic modification methyltransferase gene (coding for the C-terminal catalytic domain) and a second (unknown) gene, coding for a regulatory (N-terminal) domain^36^,^37^,^38^. A key feature of DNMT1 is its preference for hemimethylated DNA as a substrate. Hemimethylated DNA molecules (consisting of one methylated strand and an unmethylated complementary strand) are regularly produced during DNA replication (provided that the parental strands were originally methylated). High affinity of DNMT1 for hemimethylated DNA ensures the clonal propagation of methylation patterns (maintenance methylation). Other mammalian methyltransferases (like the human DNMT3A and DNMT3B) create new methylation patterns by acting on unmethylated DNA substrates (*de novo* methylation)^38^. Their N-terminal domains differ from each other and from that of DNMT1^39^.

Methylated CpG dinucleotides may silence promoters either directly, by blocking the binding of transcription factors to their recognition sequences, or indirectly, by attracting methyl-CpG binding proteins. Methyl-CpG binding proteins regularly associate with histone deacetylases and histone methylases. Removal of the acetyl moieties from histone tails and methylation of selected lysine and arginine groups results in a repressive chromatin structure silencing promoter activity^40^,^41^,^42^. These alterations may switch off active promoters and stabilize the silent status of already inactivated genes. Riggs proposed that stable gene silencing by DNA methylation may help progeny cells to „remember” their proper cellular identity, i.e. DNA methylation may provide a memory function^43^.

Transcriptional repression may depend on CpG methylation density^33^,^44^. *CpG-poor promoters* may retain their activity even if methylated. In somatic cells, a second class of promoters characterized with a moderate or intermediate CpG density (so called *„weak CpG island promoters”*) are preferentially targeted by *de novo* methylation^33^. Typical CpG island promoters (called *„strong CpG island promoters”* by Weber et al.^33^) seem to be unmethylated independent of their activity^33^ although they are regularly inactivated by DNA methylation in neoplasms^44^. Thus, focal hypermethylation seems to accompany tumorigenesis in parallel or consecutive to overall genomic hypomethylation^45^,^46^.

It is interesting to note, that an alternative system of epigenetic memory, the Polycomb-trithorax group of protein complexes, that may operate both independently from and in concert with DNA methylation, ensures a heritable regulation of gene expression via modification of histone tails, too (Bird, 2002, Cernilogar and Orlando, 2005, Laue et al., 2005). In animal cells certain sets of promoters are marked by trimethylation on lysine 27 of histone H3 (H3K27me3), a modification carried out by the enzyme EZH2. EZH2, a component of the Polycomb repressive complex 2 (PRC2) involved in gene silencing in embryonal stem cells was recently shown to attract DNA methyltransferases^47^ and may contribute, thereby, to permanent gene silencing *via* DNA hypermethylation in cancer cells^48^,^49^.

In contrast to the hypermethylated silent promoters, active promoters are usually unmethylated at CpG dinucleotides and associate with acetylated histones H3 and H4, that form *’acetylation islands’*^50^. Histone H3 di- or trimethylated on lysine 4 (H3K4me2, H3K4me3) is also enriched at active promoters. The presence of these „activating” chromatin modifications may not be sufficient, however, to ensure promoter activity because certain promoters are enriched both in „activating” and „repressive” modifications (*’bivalent’ chromatin* structure)^51^. Such *’bivalent’* domains may silence developmental genes in embryonic stem cells. In addition, Weber et al. observed that unmethylated CpG rich promoters may also associate with H3K4me2 in the absence of transcription^33^.

## Epigenetic mechanisms targeting the alternative latency promoters Cp and Qp in lymphoma cells and immortalized lympho-blastoid cell lines:

The role of DNA methylation in regulating Cp and Qp activity has been reviewed earlier (Li and Minarovits, 2003). Briefly, both Cp and Qp can be silenced, when inserted into reporter constructs, by *in vitro* CpG methylation. Accordingly, Cp is hypermethylated and silent in tumor cells corresponding to *„Cp off”* latency (including latency type I Burkitt’s lymphoma cells and latency type II Hodgkin’s lymphomas), but unmethylated and active in LCLs and the majority of posttransplant lymphomas („Cp on” latency)^24^,^52^, ^53^, ^54^. Although *in vitro* data demonstrated thatCpG methylation could silence Qp as well^55^, Qp stays unmethylated independently of its activity (i.e. it is unmethylated also in LCLs where it is switched off). Thus, DNA methylation may contribute to the silencing of Cp, but it is not used to silence Qp *in vivo*.

Episomal EBV genomes are organized into chromatin^55^. Similarly to cellular promoters^41^, ^56^, the activity of latent EBV promoters Cp and Qp also correlated with certain histone modifications marking „open” or „closed” chromatin domains^57^,^58^,^59^,^60^. In lymphoblastoid cell lines active C promoters were located to AcH3 and AcH4 rich regions^60^ that are similar to the *’acetylation islands’* characteristic to the active chromatin domains in human T cells^50^. In contrast, in type I Burkitt’s lymphoma cell lines there was no acetylation island at the inactive Cp. Active Qp, the promoter used in most cases of *„Cp off”* latency, was selectively enriched in AcH3, AcH4 and H3K4me2^59^,^60^ in type I Burkitt’s lymphoma cell lines. In lymphoblastoid cell lines, however, the inactive Qp was located to domains poor in acetylated histones.

It was demonstrated by *in vivo* footprinting that the unmethylated, active Cp was occupied by transcription factors^53^. In contrast, the absence of interactions with activating transcription factors was characteristic for highly methylated, silent C promoters. It is interesting to note, that epigenetic silencing of Cp may be regarded as a viral escape mechanism, because all of the transcripts encoding the immunodominant viral EBNA proteins are initiated at Cp. Tumor cells actively using Cp can survive, however, in severely immunsuppressed patients (e.g. B cell lymphomas in transplantation recipients and AIDS patients).

We observed that the active, unmethylated Qp was also contacted by cellular transcription factors and EBNA1, that in addition to *oriP* has binding sites at Qp as well^53^. In contrast to Cp, where silencing was correlated with the loss of transcription factor binding, silent Qp was marked by the same protein footprints as its active counterpart. A unique footprint observed only at the silent Q promoters suggested that binding of a cellular repressor might contribute to the repression of Qp activity in *„Cp on”* latency.

## Epigenetic marks at other EBV latency promoters:

*In vitro* methylation experiments demonstrated that Wp, where transcripts of EBNA1 to 6 are initiated soon after EBV infection of B cells (Figure 3), is a CpG methylation sensitive promoter^61^. Wp is switched off in LCLs, however, provided that Cp is intact, in parallel with the activation of Cp^62^. A variable methylation of Wp in LCLs suggests that epigenetic mechanisms other than CpG methylation also contribute to Wp silencing^63^.

The EBER 1 and 2 transcription units are transcribed by RNA polymerase III. Their activity is also sensitive to *in vitro* CpG methylation that blocks binding of the nuclear proteins c-myc and ATF to the 5′region of EBER1p^64^. EBERs are expressed in the major EBV-carrying cell types and the EBER locus is hypomethylated^65^.

LMP1 expression can be related to the methylation status of the LMP1 promoter. In type I BL lines LMP1p is silent and highly methylated, whereas in LCLs and midline granulomas it is unmethylated and active (reviewed by Li and Minarovits, 2003). In contrast to Cp, LMP1p activity did not correlate with transcription factor occupancy^53^.

In lymphoid cell lines, expression of LMP2A seems to be regulated by the combinatorial effects of DNA methylation, histone acetylation and the level of histone H3 dimethylated on lysine 4 (H3K4me2, a marker of „open” chromatin)^66^.

The promoter for the BARTs (also called CSTs including BARF0) RNAs was found to be active and unmethylated in the only nasopharyngeal carcinoma cell line it was looked for^67^.

## Latent Epstein-Barr virus proteins interacting with the cellular epigenetic regulatory machinery:

We demonstrated above that various epigenetic control mechanisms leave cell type specific marks on latent EBV genomes, establishing thereby distinct *viral epigenotypes* (reviewed by Minarovits,^68^). In turn, certain EBV encoded proteins may interact with the cellular epigenetic regulatory machinery, affecting thereby the activity of a set of cellular promoters (Table 3).

Because EBNA1 has binding sites in the human genome^69^ and it is known that high affinity EBNA1 binding can cause site specific demethylation within *oriP*, the latent origin of EBV replication^70^, we suggest that *binding of EBNA1 to its cellular target sites may elicit local demethylation and contribute thereby to the activation of silent cellular promoters*. Lin et al. demonstrated in model experiments that high affinity of the binding protein and protein binding site occupancy is crucial in targeted demethylation^71^.

EBNA2, the major viral transactivator protein expressed in *„Cp on”* latency, may switch on both viral (Cp, LMP1p, LMP2Ap) and cellular promoters (CD21p, CD23p, AML-2p, BATFp, IL-16p) (reviewed by Györy and Minarovits^72^). EBNA2 activates its target promoters through binding to CBF1 (C promoter binding factor 1), a cellular protein that also interacts with the activated cellular Notch family of proteins^73^–^76^. *In vivo* footprinting confirmed CBF1 binding to the active C promoter and LMP2A promoter but not to their inactive counterparts^53^,^77^. In addition to CBF1, EBNA2 also associates with the cellular histone acetyltransferases p300, CBP, and PCAF^78^. Wang et al.^78^ speculate that these histone acetyltransferases may enhance the ability of EBNA2 to up-regulate expression of the viral oncogene LMP1 by counteracting the effect of histone deacetylases associated with the silent LMP1 promoter^78^.

EBNA3C (EBNA6), a nuclear antigen essential for EBV-dependent immortalization of B cells may fine tune (down-modulate) the activity of EBNA2-activated promoters by interacting and competing with prothymosin alpha, a partner of p300^79^,^80^. In addition, EBNA3C may also form complexes with histone deacetylases (HDAC1 and 2) in human B cells^81^. Knight et al^81^ proposed that EBNA3C may be in separate complexes with histone acetylases and deacetylases affecting the function of distinct viral and cellular promoters.

In contrast to EBNA3C, the partly nuclear, parly cytoplasmic antigen EBNA-LP (EBNA leader protein, EBNA5) strongly up-regulates EBNA2-mediated transcription by relocalizing the histone deacetylase HDAC4 from the nucleus to the cytoplasm^82^. Based on their observations Portal et al.^82^ suggested that the coactivator function of EBNA-LP is due to the reduction of the HDAC4 concentration in the nucleus. They also speculated that EBNA-LP coactivates with EBNA2 by displacing HDAC4 (and HDAC4 associated repressors) from EBNA2-bound promoters. Recent data do not support, however, the nucleocytoplasmic model for EBNA-LP mediated LMP1p coactivation, because an EBNA-LP isoform, defective in shuttling to the cytoplasm, could coactivate EBNA2 and associate with HDAC4^83^.

LMP1, a transmembrane protein involved in malignant transformation can affect both alternative systems of epigenetic memory, DNA methylation and the Polycomb-trithorax group of protein complexes. Tsai et al. observed, however, that expression of the EBV-encoded oncoprotein LMP1 (latent membrane protein 1) in epithelial cells up-regulated the expression and activity of cellular DNA methyltransferases 1, 3a and 3b *in vitro*^84^. This resulted in hypermethylation of the E-cadherin promoter and down-regulation of E-cadherin gene expression. LMP1 activated DNMT1 via the c-jun NH(2)-terminal kinase/activator protein-1 (JNK-AP-1) signaling pathway^85^. In addition, formation of a transcriptional repression complex (composed of DNMT1 and histone deacetylase) could be detected on the E-cadherin promoter as a consequence of LMP1 action. How this repressor complex was targeted to the E-cadherin promoter, however, remains to be elucidated. These results observed in epithelial cells are certainly very much relevant to the development of EBV-associated lymphomas as well, because latent EBV infection apparently results in hypermethylation of a set of cellular promoters not only in nasopharyngeal carcinoma^86^ and EBV-associated gastric carcinoma^87^,^88^, but also in typically LMP1 positive Hodgkin lymphomas^89^,^90^,^91^, iatrogenic lymphomas^92^, and lymphomas developing in AIDS patients^93^.

Although the oncoprotein LMP1 can up-regulate the PcG protein Bmi-1^94^, there are no data regarding the contribution of PcG (or trithorax group) proteins to the regulation of latent EBV promoters. Up-regulation of Bmi-1 is mediated by NF-κB signaling both in EBV-positive and EBV-negative Hodgkin’s lymphoma cells. Dutton et al.^94^ suggested that by activating NF-κB, and thereby up-regulating Bmi-1, LMP1 may contribute to the loss of B-cell identity in EBV carrying Hodkins’s lymphomas *via* down-regulating a series of B-cell markers (CD21/MS4A1, BLK, LY9). In EBV-negative disease other activators of NF-κB may substitute for LMP1. We speculate that Bmi-1, as a component of polycomb repressor complex 1 (PRC1), may act by marking a set of promoters for *de novo* methylation by DNA methyltransferases. In addition, Bmi-1, using the same mechanism, may down-regulate a series of tumor suppressor genes (including IGSF4 and ATM), too. Bmi-1 also mediates up-regulation of certain genes that are transcriptional targets of LMP1 (*STAT1* and *c-MET* and *HK*). These genes code for signaling molecules and a hexokinase maintaining high glycolytic activity and seem to play an important role in lymphomagenesis.

It is worthy to mention that LMP1 may affect the levels of certain cellular microRNAs (and modulate thereby cellular gene expression programs) by activating the NF-κB pathway. LMP1 induced expression of miR-146a, a microRNA that may affect the interferon response (Cameron et al., 2008), and elevated the level of miR-155, a microRNA implicated in the development of B cell lymphomas 95.

## Stem cell chromatin patterns in B-cell lymphomas:

Burkitt lymphomas (BLs) and diffuse large B-cell lymphomas (DLBCLs) seem to share a set of *de novo* methylated genes (e.g. *HOXB13, CALCA, NEFL, PROK2*) that are repressed by the polycomb repressive complex 2 (PRC2) in embryonic stem cells, in spite of the differences of BLs and DLBCLs regarding morphology, genetic background, and transcriptional pattern^96^. The aberrant DNA methylation observed in lymphomas was absent in adult stem and progenitor cells. Based on these data Martin-Subero et al.^96^ suggested that BLs and DLBCLs may originate from cells with stem cell features or acquire such features during lymphomagenesis by epigenetic remodeling.

## Tumor specific and EBV-associated gene expression and CpG methylation changes in lymphomas:

The epigenetic alterations of the host genomes in EBV-associated neoplasms (lymphomas and carcinomas) were reviewed recently^97^.

It is obvious that certain common tumor suppressor and tumor-associated genes were found to be silenced by DNA methylation in more than one lymphoma types in independent studies. One could also discern, however, methylation profiles that seem to be unique for individual lymphoma types. The apparently specific contribution of EBV to the alteration of gene expression in HD biopsies^98^ remains to be correlated with the corresponding epigenetic changes. Elucidation of the temporal sequence of genetic events (e.g. the Ig/c-myc translocation in BL), epigenetic alterations (e.g. stem cell-like epigenetic marks of BL cells) and EBV infection (an early event during the genesis of BL) will be indispensable in case of each EBV positive lymphoma type for a better understanding of lymphoma development and progression. In addition, the patho-epigenetic changes induced by EBV, similarly to those elicited by a growing number of other viruses and certain bacteria may offer new opportunitis for therapeutic intervention.

## Figures and Tables

**Table 1.** Major EBV associated neoplasms (for details see Niller et al, 2007 and 2008).

***Lymphomas*** Burkitt’s lymphoma Hodgkin’s disease T/NK-cell lymphoma X-linked lymphoproliferative disease Posttransplant lymphoproliferative disease AIDS-associated lymphoma Lymphomas in metothrexate-treated rheumatoid arthritis and polymyositis patients
***Carcinomas*** Nasopharyngeal carcinoma Gastric carcinoma Carcinomas of the major salivary glands Thymic carcinoma Mammary carcinoma
***Sarcoma*** Leiomyosarcoma

**Table 2.** Major latency types of Epstein-Barr virus

**Latency type**	**Representative cell type**	**Expressed products**
***„Cp off latency”***		
Type 0 latency	Resting B cell	EBNA1 (variable ?), LMP2A (?)
Type I latency	Burkitt’s lymphoma	EBNA1, EBER1 & 2, BART, microRNAs
Type II latency	Hodgkin’s lymphoma	EBNA1, EBER1 & 2, BART, LMP1 & 2, microRNAs
	Nasopharyngeal and gastric carcinoma	EBNA1, EBER1 & 2, BART, LMP1 (variable expression), LMP2, microRNAs, BARF1
***„Cp on latency”***		
	Lymphoblastoid cell lines	EBNA1-6, EBER1 & 2, BART, LMP1 & 2, microRNAs

**Table 3.** Latent Epstein-Barr virus products interacting with the cellular epigenetic regulatory machinery

**Latency product**	**Cellular partner**	**Putative outcome of the interaction**
**EBNA1**	EBNA1 binding site	*site-specific demethylation of DNA; activation of adjacent genes;*
**EBNA2**	histone acetyltransferases (p300, CBP, PCAF)	*transactivation of genes;*
**EBNA3C (EBNA6)**	prothymosin alpha, p300; histone deacetylases (HDAC1 & 2)	*modulating EBNA2-mediated transactivation*
**EBNA-LP (EBNA5)**	histone deacetylase 4 (HDAC4)	dispacement of HDAC4 from EBNA2-activated promoters; *coactivation*
**LMP1**	DNA methyltransferases DNMT1, 3a, 3b;	hypermethylation mediated *silencing of promoters;*
	Bmi-1 (a component of PRC1);	*silencing* of B cell specific and tumor suppressor genes;
		*marking* a set of promoters for *de novo methylation by DNMTases;*
		*up-regulation of transcription (STAT-1, c-MET, HK);*
	*via* the NF-κB pathway: microRNA coding genes	alteration of miR-146a and miR-1 level; *modulation of cellular mRNA levels*

**Table 4.** Cellular genes silenced by promoter hypermethylation in EBV-associated lymphomas and derived cell lines. For detailed discussion see Niller et al.^97^

**Gene**	**Lymphoma**		
	**BL**	**HD**	**Iatrogenic/AIDS-associated**
ABF1	+	−	
BCL2-family (proapoptotic members)	+		
BCMA	+		
BOB.1/OBF.1	+		
CHK2 kinase		+	
Cyclin D2	+		
CD19		+	
CD20		+	
CD79B	+		
CD59/HRF20	+		
DAPK	+	**±**	+
DLC1	+	**±**	
FHIT	+		
GADD25G	+		
GSTP1	+		
LCK		+	
MAPK10/JNK3	+	**±**	
O6-methylguanine-DNA MTase			+
p16/CDK4A	+	**±**	−
p15/CDK4B	+	**±**	−
p57/KIP2	+		
p73	+		
PCDH10	+	+	
PLK2	+		
PU.1		+	
PTPN13/FAP1	+		
RASSF1A	−	+	−
SYK		+	
WNT5A	+		

BL: Burkitt’s lymphoma; HD: Hodgkin’s disease; iatrogenic lymphomas include post-transplant lymphoproliferative disorders (PTLDs) and methotrexate-related lymphomas.

Symbols: frequent inactivation by promoter methylation, +; infrequent inactivation by promoter methylation, −/+; typically unmethylated promoter, −.
